# Combination of High Dose Hypofractionated Radiotherapy with Anti-PD1 Single Dose Immunotherapy Leads to a Th1 Immune Activation Resulting in a Complete Clinical Response in a Melanoma Patient

**DOI:** 10.3390/ijms21186772

**Published:** 2020-09-15

**Authors:** Clara Milhem, Olivier Moralès, Céline Ingelaere, David Pasquier, Serge Mordon, Laurent Mortier, Xavier Mirabel, Nadira Delhem

**Affiliations:** 1 INSERM, CHU-Lille, U1189—ONCO-THAI—Assisted Laser Therapy and Immunotherapy for Oncology, University of Lille, F-59000 Lille, France; clara.milhem@immune-insight.com (C.M.); olivier.morales@ibl.cnrs.fr (O.M.); serge.mordon@inserm.fr (S.M.); 2 Immune Insight, Institut de Biologie de Lille, 59021 Lille, France; ingelaereceline@gmail.com; 3 CNRS UMS 3702, Institut de Biologie de Lille, 59021 Lille, France; 4 Service de Radiothérapie, Centre Oscar Lambret, 59000 Lille, France; d-pasquier@o-lambret.fr; 5 Service de Dermatologie, Hôpital Huriez, 59000 Lille, France; Laurent.MORTIER@CHRU-LILLE.FR

**Keywords:** stereotaxic radiotherapy, immunotherapy, anti-PD1, melanoma, immune response

## Abstract

The development of immunotherapy has recently modified the anti-tumor therapeutic arsenal; particularly, immune checkpoint inhibitors have led to a significant increase in overall survival. The current challenge is now to select good responder patients by identifying early biomarkers to propose therapeutic combinations that potentiate the efficacy of the therapy. Here we report the case of a 60-year-old man with superficial melanoma treated with high-dose hypo fractionated radiotherapy (H-SRT) combined with a single dose of anti-PD1 immunotherapy (Nivolumab) for a metastatic lymph node recurrence due to cancer progression. In this study, we present the results obtained regarding the activation of the Th1 immune response after H-SRT treatment followed by anti PD-1 therapeutic protocol. These results were correlated with clinical data to identify potential immunological biomarkers of treatment efficacy. This exceptional case report shows that a combination of H-SRT with a single dose of anti-PD1 immunotherapy may allow a better activation of the immune response in favor of a complete clinical response.

## 1. Introduction

Melanoma is the most common lethal skin cancer form. For malignant melanoma, most cases are diagnosed at an early stage, when surgical excision can be curative. However, some patients can already be at a metastatic stage at diagnosis, and some develop metastases after their initial treatment. Radiation therapy is one of the therapeutic options that can be used to treat it or as an adjuvant way to combat it [1].

Cancer cells are no longer exclusively characterized by genomic alterations’ acquisition, but also by their ability to escape immune surveillance [2]. Indeed, the anti-tumor immune response develops in three successive phases, the so called 3E theory redefined by Dunn et al. The Elimination phase is the first one during which immune cells recognize and manage tumor cells’ elimination. The second one is the Equilibrium phase during which the persistent tumor cells are dormant or continue to accumulate genetic abnormalities. Furthermore, finally, the Escape phase when the tumor overpasses the immune system, proliferates and spreads [3,4].

Recently radiotherapy protocols combining high dosed sessions over a shorter total treatment duration have emerged and are called high dose hypofractionated radiotherapy (H-SRT). Stereotactic irradiation techniques allow better targeting of the tumor, thus making it possible to administer higher doses of ionizing radiation [5]. Preclinical data suggest that high doses per fraction generate a superior immunological response, responsible for an abscopal effect [6]. Described for the first time in the 1950s, the abscopal effect is characterized by tumor regression at an irradiated distance site [7]. The mechanisms of this process are still unclear, but greater antigens and danger signals release (as Calreticulin, damage-associated molecular pattern molecules, high-mobility group box 1 and adenosine triphosphate) would allow the activation of the immune system [8].

Metastatic melanoma treatment has recently been improved by targeted therapies and immunotherapy progress. Most tumor cells express antigens recognizable by the immune system. Nevertheless, some mechanisms permit tumors to escape immunosurveillance such as regulatory T cells and immune check point (ICP) activation.

ICP (CTLA-4, PD-1, PD-L1 among others) can be blocked by immune checkpoint inhibitors (ICI). By blocking the brakes, immune system reactivation becomes possible and tumor cell effective targeting is allowed. Such strategies rely on specific monoclonal antibodies (mAb) used to specifically target and inhibit ICP. Anti-PD-1 and the anti-PD-1 ligand (PD-L1) are the most used mAb in melanoma. In fact, unlike normal cells, cancer cells express more often PD-L1 [9] and PD-1 is also expressed on Tregs in association with tumor escape [10,11]. Therefore, blocking these ICI would restore a good immune response, that will be fundamental to cancer treatment.

There are an increasing number of studies indicating that combining radiotherapy with immunotherapy would stimulate the abscopal effect [12]. Indeed, radiation therapy is a particularly promising candidate for association with the ICI [13]. In fact, RT effectiveness is well established; it is an accessible and relatively economical procedure with limited and manageable side effects. It is now clear that RT has multi-level immunomodulatory effects. Those effects, which emerge locally in the irradiated field, also have a systemic impact and can be exploited to boost the therapeutic activity of systemic treatments. Some of these effects have been identified as able to increase antigen presentation, NK cell activation and IFN release. H-SRT seems to have a higher immunostimulatory effect than standard RT, particularly in inducing a Th1 response [13]. Clinical trials are underway to validate this association [14], but there is no integrated analysis of underlying cellular, molecular and systemic mechanisms.

Other recent advances confirm the interest of a synergy between radiotherapy and immunotherapy via the abscopal effect [15,16]. One of the first studies of anti-PD-1 (Nivolumab©) and radiotherapy combination in patients with metastatic melanoma showed a 46% response rate on non-irradiated lesions. Irradiated lesion response rate was 44% for those who received sequential RT and then PD-1 treatment, and 64% for those who received concomitant treatment [17]. However, the extent to which radiation therapy is involved in these distant responses is not yet known.

In this context, this case report study aims to evaluate the regulation of the immune response upon H-SRT followed by anti PD-1 therapeutic protocol and to correlate immunological and biological data with clinical outcomes in order to identify potential biomarkers of Th1 immune activation and treatment efficacy.

## 2. Case Presentation

### 2.1. Patient’s Outcome after High Dose Hypofractionated Radiotherapy

The patient is a 60 year-old-man, monitored since 2003 for a superficial spreading melanoma on the right leg, firstly treated by surgical excision and lymph node dissection. 

The lesion was not BRAF mutated and was classified as high risk with a level IV of Clark, lymph node involvement and with a Breslow index of 1.8-mm. Histological analysis did not show ulceration. Right inguinal sentinel lymph node analysis highlighted micrometastases on the three removed nodes (3N+ CR-/3N). The patient undergoes lymph node dissection (5N-/5N). He received Interferon-α adjuvant therapy for 18 months.

**Figure 1:**
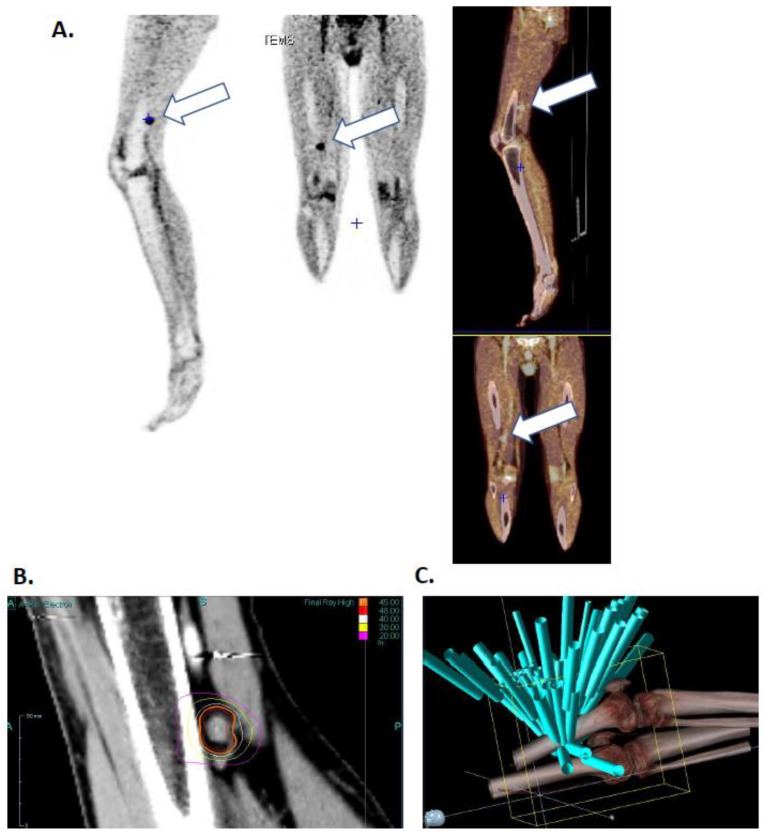
Patient’s right leg positron emission tomography (TEP) image (17/02/2016) (**A**), image of isodose curve around the lesion (**B**) and visualization of the 65 irradiation ray (**C**) White arrow point patients irradiated tumor.

He presented two skin recurrences in 2011 and 2015 each treated by surgical excision. In 2016, he presented a third recurrence with popliteal lymphadenopathy (Figure 1A) with radiotherapy indication. He was then included in the EarlyBio clinical trial (ClinicalTrials.gov Identifier: NCT02439008) in March 2016.

The patient’s lesion was targeted with high dose hypo fractionated radiotherapy (15 grays), three times during one-week (March 11th, 13th and 17th) using the Cyberknife (Accuray, Sunnyvale, CA, USA) robot in the Centre Oscar Lambret anti-cancer center (Lille). Figure 1B shows isodose curves surrounding the lesion and Figure 1C shows the 65-irradiation rays. Both figures highlight a high radiation impact on the lesion as well as an excellent healthy tissues protection. Days following irradiation the patient presented chills and vitiligo, symptomatology that he had already presented during interferon alpha treatment.

Clinical and imaging evaluations were performed 3 months after the end of radiotherapy, the patient presented a dissociated response with regression of the irradiated lesion but a progression of a subcutaneous nodule, not visible at the imagery but palpable under the skin. 

### 2.2. Complete Clinical Response after Combination of High Dose Hypofractionated Radiotherapy with Anti-PD1 Single Dose Immunotherapy 

A treatment change was decided, and he received a first injection of Nivolumab (OPDIVO^®^ Bristol-Myers Squibb) at a dose of 3 mg/kg, administered as a 60-min intravenous infusion, shortly after (June 2016). 

Sub-cutaneous metastasis excision was programmed in July 2016, but, unexpectedly, the patient presented a complete response after this single injection; the surgery was then cancelled. The treatment was not renewed due to poor tolerance (acute polyarthritis stage 3 development). Clinical and imaging evaluations showed a complete response at 9 and 12 months after treatment with strong decrease of fixation on the irradiated lymphadenopathy and disappearance of right thigh suspicious subcutaneous fixation. He did not receive any new treatment since then. The patient’s last TEP (March 2020) did not show any abnormal fixation meaning that total response is still maintained 4 years after the last treatment.

### 2.3. Peripheral Immune Cell Activation after Combination of High Dose Hypofractionated Radiotherapy with Anti-PD1 Single Dose Immunotherapy 

Blood samples were collected on dry and EDTA tubes according following the following timeline: before treatment (T0), 15 min after H-SRT session (T1, T2, T3), one week (T4), 3, 6, 9 months (T4, T5, T6, T7) and one year (T8) after the last sequence of H-SRT.

Peripheral blood mononuclear cells (PBMCs) were isolated by density gradient from fresh blood collected in two EDTA tubes (Greiner Bio One Vacuette^®^, Les Ulis, France) using lymphocyte separation medium (Eurobio, Les Ulis, France). The PBMC ring was recovered after centrifugation and PBMCs were washed two times in PBS (GIBCO, Life Technologies, Paisley, UK). 

After counting, at least 4 million of PBMCs were studied by flow cytometry using FACS Canto II flow cytometer powered by FacsDiva software (BD Biosciences, Franklin Lakes, NJ, USA). PBMCs were split in 4 panels to be stained with fluorochrome-conjugated mAbs or corresponding isotype control mAbs as describe in Table 1 (Miltenyi Biotec, Bergisch Gladbach, Germany). Data analysis was made using FlowJo software (ACEA Biosciences, San Diego, CA, USA). Results were presented as population frequency or as median fluorescence intensity (MFI) using Prism 8 (Graphpad Software Inc, San Diego, CA, USA).

**Figure 2:**
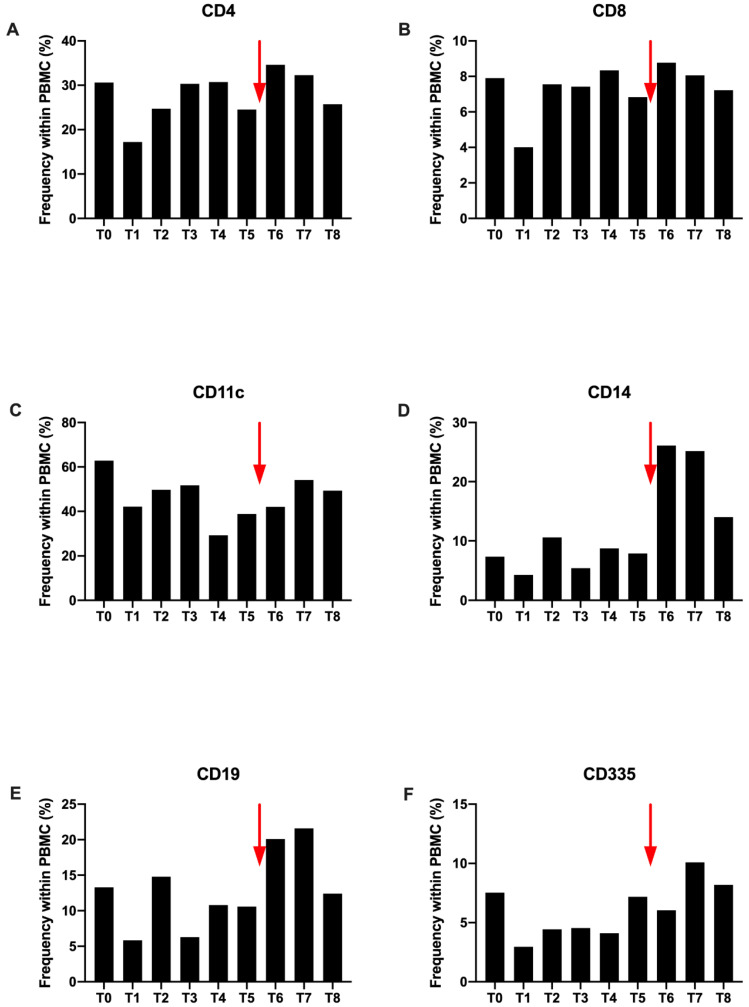
Expression of CD4+ (**A**), CD8+ (**B**), CD11c+ (**C**), CD14+ (**D**), CD19+ (**E**) and CD335+ (**F**) markers among the patient’s peripheral blood mononuclear cells. All these results were obtained from flow cytometry assay and expression percentage. The red arrow corresponds to the single injection of Nivolumab. T0 to T8 correspond to all the study time points as described above from before the radiotherapy to 1 year after the last session.

Overall, after the first H-SRT session (T1), a decrease is observed in different immune cell sub-populations compared to T0. However, their frequencies increase at T2 and remain stable until T4 (Figure 2).

After the Nivolumab introduction (T6), most of the immune sub-populations continue to increase. This is more important for CD4+, CD14+ and CD19+ (Figure 2A,D,E). This increase is found at T7, with a higher proportion for CD11c+ dendritic cells. At the end of the study all populations decrease with a return to proportions comparable to those observed at T0, except for a larger CD14+ monocyte population (Figure 2).

As shown for the other immune cells, CD4+ T cell frequency decreases after the first session of H-SRT (T1), from 31% to 17%. Then, it increases again from T2 to T4, to come back to the steady state. When the disease is progressing (T5) CD4+ population drop to 25%. Immediately after Nivolumab injection, the frequency increases again, (35%) and gradually decreases until T8 (Figure 2A). A similar pattern is observed for CD8+ cells (Figure 2B) and CD11c+ cells (dendritic cells and B cells) (Figure 2C). For CD14+ cells (monocytes), the variation is different during radiotherapy with a decrease at T1, then an increase at T2 and a decrease at T3. However, after T5 and Nivolumab injection, the evolution of the profile is the same (Figure 2D). For CD355+ cells (NK cells), the only difference observed with CD4+ cells is a decrease at T6, with a more delayed increase, at T7 after Nivolumab injection (Figure 2F).

**Figure 3:**
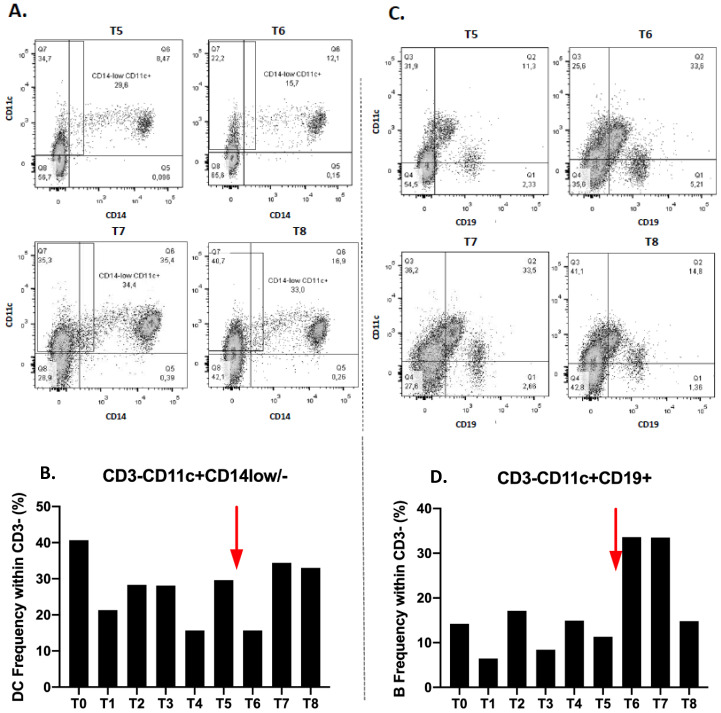
Gating strategy for frequency of CD11c+CD14low/- dendritic cells (**A**) and CD11c+CD19+ B lymphocytes (**C**) before (T5) and after (T6–8) Nivolumab injection (red arrow). Frequency of CD11c+CD14low/- dendritic cells (**B**) and CD11c+CD19+ B lymphocytes (**D**) in CD3-cells during all the follow up.

Dendritic cells subset (CD3-CD11c+CD14low/-) also decreases at T1, increases again at T2 and stabilizes at T3 (28.1%). There is a noticeable decrease at T4 and a re-increase at T5 (Figure 3B). After immunotherapy treatment, their frequency decreases immediately to T6 then increases to T7 and stabilizes at T8 (Figure 3A,B).

We also observed a decrease of the B lymphocyte fraction (CD3-CD11c+CD19+), at T1 and then an increase at T2 and a decrease at T3. Their proportion therefore varies during radiotherapy. At T4, the rate increases and decreases again at T5 (Figure 3D). After the anti-PD1 introduction, there is a significant increase in the level of lymphocyte B to T6 which remains at T7. Their proportion decreases again to T8 (Figure 3C,D).

We also observed a decrease of the B lymphocyte fraction (CD3-CD11c+CD19+), at T1 and then an increase at T2 and a decrease at T3. Their proportion therefore varies during radiotherapy. At T4, the rate increases and decreases again at T5 (Figure 3D). After the anti-PD1 introduction, there is a significant increase in the level of lymphocyte B to T6 which remains at T7. Their proportion decreases again to T8 (Figure 3C,D).

**Figure 4:**
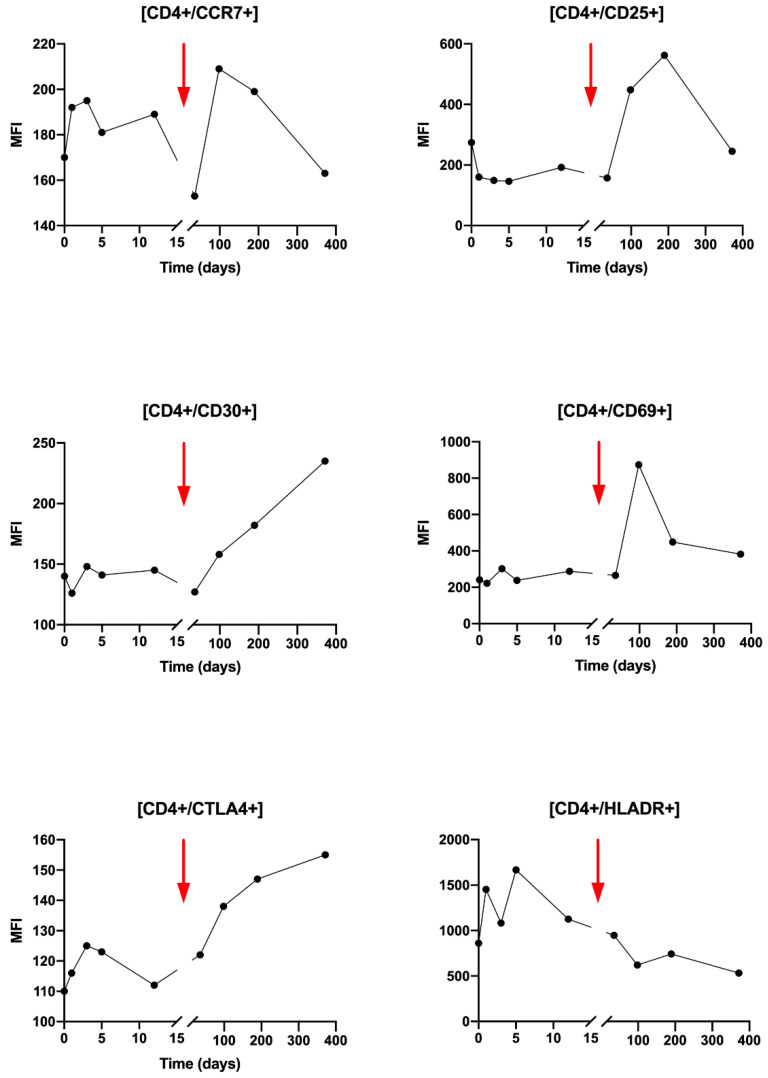
Analysis of CD4+ T lymphocytes activation state by flow cytometry for several activation markers: CCR7, CD25, CD30, CD69, CTLA4, HLADR. Results are presented over time (days) and expressed in median of fluorescence (MFI). The red arrow points the Nivolumab injection timepoint.

Regarding the activation status of the CD4+ T cells during radiotherapy, slight variations in activation markers are observed, except for HLA-DR which increases from T0 to T3 (Figure 4). Very interestingly, after Nivolumab injection, a clear increase in CD25, CD30, CD69 and CTLA-4 activating markers was observed (Figure 4). An increase is also found for CCR7 but less importantly. Conversely, the HLA-DR marker decreases from T5 to T8.

**Figure 5:**
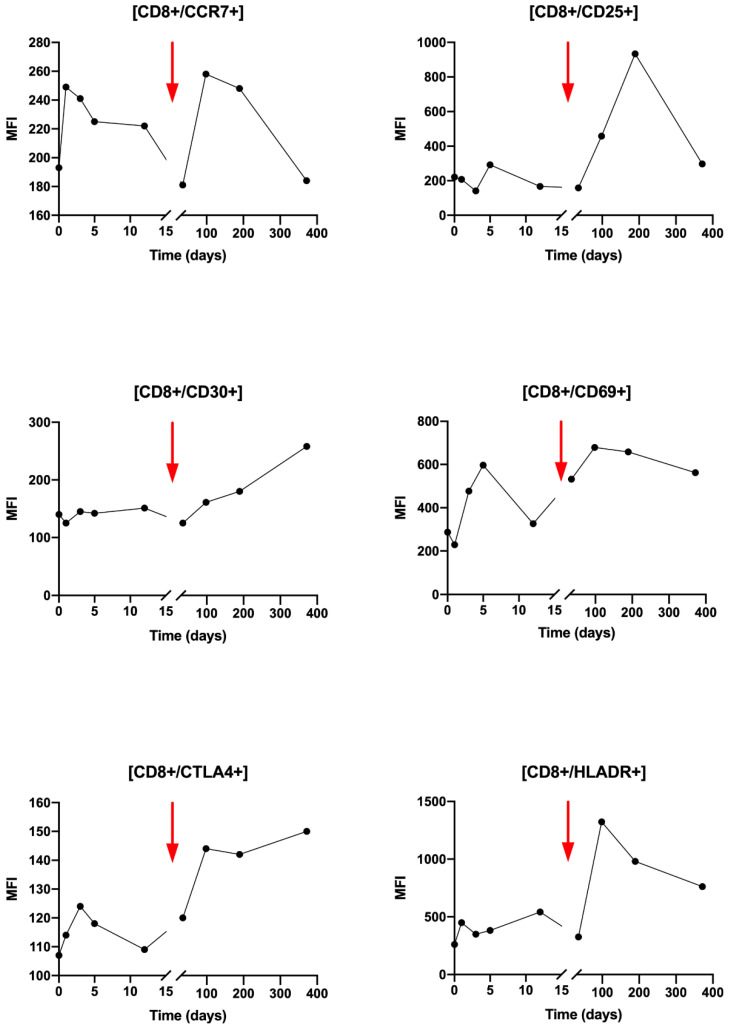
Analysis of CD8+ T lymphocytes activation state by flow cytometry for several activation markers: CCR7, CD25, CD30, CD69, CTLA4, HLADR. Results are presented over time (days) and expressed in median of fluorescence (MFI). The red arrow points the Nivolumab injection timepoint, 90 days after the beginning of the follow up.

Regarding CD8+ T cells activation, during T0 to T3, small variations were observed for the CCR7, CD25, CD30, CTL4 and HLA-DR markers (Figure 5). Compared with CD4+ LT, the observed changes are greater after Nivolumab injection after T5 with an increase in all markers: CCR7, CD25, CD30, CD69 and HLA-DR. It can be noted that the variations of the CCR7 marker are similar after the beginning of the radiotherapy and the Nivolumab treatment.

**Figure 6:**
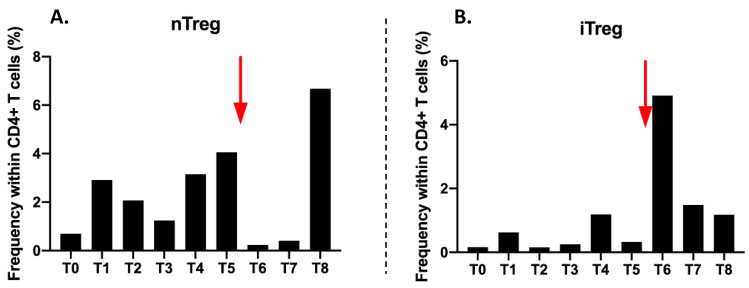
Frequency (in percentage) of CD4+CD25+/highCD127-/low nTreg (**A**) and frequency of CD4+CD18+CD49b+iTreg lymphocytes in CD4+ cells (**B**) during all timepoints of the follow up. The red arrow represent the time of the single Nivolumab injection after T5.

The proportion of CD4+CD25highCD127-/low nTregs in CD4 T cells is very low (0.7%) before treatment (T0). There is an increase to 2.91% at T1 and then a decrease from T2 to T3. Their proportion remains low during radiotherapy. Then it increases from T4 to T5 at 3.15% and 4.06%, respectively. After Nivolumab injection, nTregs collapse at 0.23%. A very large increase at T8 at 6.68% at the end of follow-up is observed (Figure 6A).

The proportion of induced Treg (iTregs) is very low at 0.16% at T0. It varies during radiotherapy from T0 to T3, but in values that remain very low. The major change is observed at T6 with an increase to 4.91% just after the injection of Nivolumab. Then the proportions decrease gradually until T8 to reach 1.18% (Figure 6B).

### 2.4. Activating Cytokine Production after Combination of High Dose Hypofractionated Radiotherapy with Anti-PD1 Single Dose Immunotherapy 

Patient’s sera were collected after coagulation in dry tubes (Greiner Bio One Vacuette^®^, Les Ulis, France). Sera were stored at −80 °C until the entire sera collection was complete. Enzyme-Linked ImmunoSorbent Assay (ELISA) was used to detect and quantify sera for 8 cytokines (IL10, IL12, IFN-γ, TGFβ, IL4, IL6, IL2 and TNFα) (BD Pharmingen, Franklin Lakes, NJ, USA) following manufacturer’s recommendations. Readings were made at 492 nm using the Multiskan spectrometer (Multiskan EX, ThermoLabsystems, France) powered by Ascent Software (ThermoFisher Scientific™, Walthman, MA, USA). 

**Figure 7:**
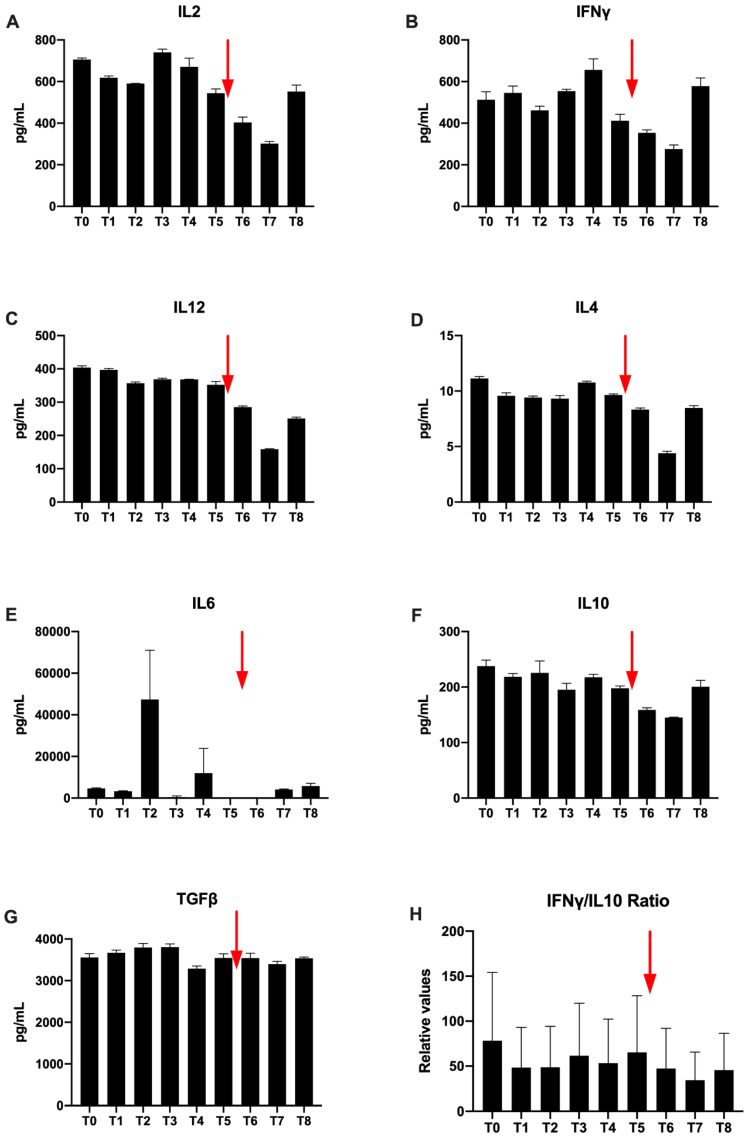
Secretion of IL2 (**A**), IFNγ (**B**) and IL12 (**C**) Th1 cytokines, IL4 (**D**) and IL6 (**E**) Th2 cytokines and IL10 (**F**) and TGFβ (**G**) immunosuppressive cytokines in patient’s sera. represented in pg/mL. Representation of IFNγ/IL10 ratio (relative values) (**H**). The time of the Nivolumab single dose injection is represented by the red arrow.

Regarding Th1 cytokines, IL-2 concentrations are higher than 700 pg/mL at T0. Variation are observed during radiotherapy. A spontaneous decrease is observed at T5, which increases after Nivolumab injection and the concentration increases again at T8 (Figure 7A). Furthermore, for IFNγ, a high T0 concentration of 547 μg/mL is also observed. Then, there is a profile similar to IL2 with a slight decrease followed by an increase during radiotherapy (Figure 7B). At T5, the concentration decreases. The concentration increases again to T8. No value for TNFα was detected in ELISA.

Il-12 is a cytokine predominantly secreted by activated dendritic cells. Its concentration is raised to 404 pg/mL at the beginning of the treatment and remains stable during the radiotherapy treatment. On the other hand, there is a decrease after the injection of Nivolumab until T7 (158 μg/mL) and a final slight increase at T8 (Figure 7C).

Regarding Th2 cytokines only a low IL-4 concentration is detected (Figure 7D). Nevertheless, by looking at the overall evolution, Th2 cytokines are similarly observed and the concentrations decrease after Nivolumab injection. The concentration finally increases again to T8. However, in the immediate aftermath of T4 and T6 radiotherapy, there was no detectable IL-6 (Figure 7E). Values increase again at T7 and T8 to find respective values at 1760 μg/mL and 2240 μg/mL, higher than T0 (Figure 7E).

Regarding, immunosuppressive cytokines, the concentrations vary faintly during radiation therapy (Figure 7F,G). Before H-SRT TGFβ, concentration is very high (3550 μg/mL) (Figure 7G). It increases during radiotherapy. Then, a concentration decrease is observed. Concentrations vary only slightly between T5 and T6. The last TGFβ concentration is equivalent compared to T0 (Figure 7G). For IL10, a spontaneous decrease is observed at T5 at 148 pg/mL, which increases after Nivolumab injection from T6 to T7 (Figure 7F). 

Finally, the IFNγ/IL-10 ratio, used in immunology to compare anti-tumor versus immunosuppressive cytokines, is in favor of the anti-tumor cytokine (IFNγ). This ratio is not changed during radiotherapy. It increases gradually after the Nivolumab injection at the end of follow-up; from T7 to reach a final value greater than 5 to T8 (Figure 7H).

### 2.5. Modulation of the Effect of the Patient’s Exosomes on Immune Cell Proliferation After Combination of High Dose Hypofractionated Radiotherapy with Anti-PD1 Single Dose Immunotherapy 

Exosomes were isolated from plasma after several centrifugation and ultracentrifugation. They are selected using a sucrose cushion as described in a previous publication [18]. After isolation exosomes were stocked at −80 °C. Exosomes were then dosed using the colorimetric Bradford method (BIORAD Laboratories, Hercules, CA, USA). For functional assay PBMC were activated by 1 µg/mL of Phytohemagglutinin (PHA) (Sigma, St Louis, MO, USA) before to be co-incubated with 3.33 µg/mL of the patient’s exosomes.

After 72 h of total incubation with exosomes, PBMC proliferation was analyzed after 18 h of incubation with [methyl-3H]-thymidine (1 μCi/well) (PerkinElmer, Waltham, MA, USA). The incorporation was evaluated using a 1450 Trilux luminometer (Perkin Elmer, Walthman, MA, USA) after the filtration of plates in a glass fiber filter (Perkin Elmer, Walthman, MA, USA) and the addition of liquid scintillation (Beckman, Brea, CA, USA). Results are the mean of triplicates and are expressed in count per minute (CPM). 

**Figure 8:**
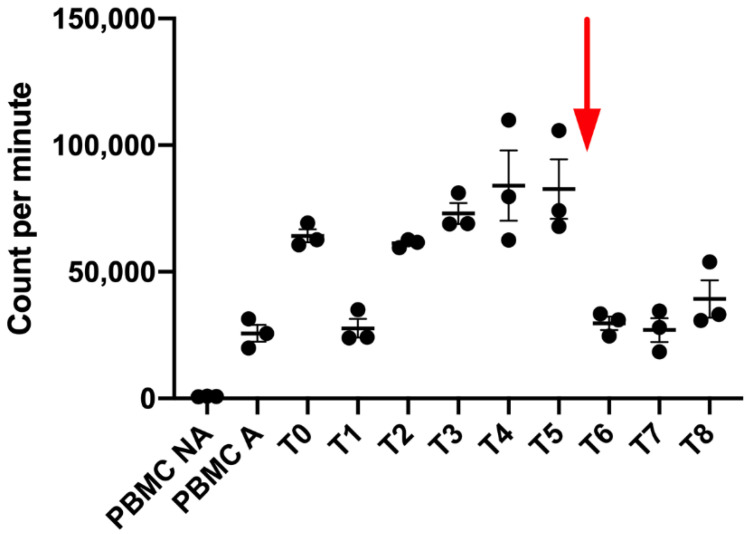
Patient’s Exosomes effect on regulation of activated PBMC (PHA 1 µg/mL). Activated PBMC only (PBMC A) use as positive control and non-activated PBMC (PBMC NA) as negative control. The red arrow represent time of the single dose injection of Nivolumab.

After HRT, we first observed at T1, T2 and T3 a decrease of the PBMC proliferation when they were co-cultured with exosomes isolated from the melanoma patient, followed by an increase of this proliferation since T3 to T6. However, just after Nivolumab injection, we observed a shift of the exosome impact on immune cells (Figure 8). Indeed, we clearly observed after the Nivolumab treatment a decrease of the immune activating potential of the newly produced exosomes. 

## 3. Discussion

Immune checkpoint inhibitors (ICIs) have revolutionized cancer treatment. Nivolumab (anti-PD1 monoclonal antibody) has been shown to be effective in metastatic melanoma treatment. However, despite the increase in overall survival in 33–40% of melanoma patients, anti-PD1 treatment did not show efficacy in the majority of treated patients [19,20]. Indeed, the effectiveness of these treatments varies greatly depending on the individual and the type of cancer. This raises the question of therapeutic combinations, particularly with radiotherapy, which would be rationally able to modify the immunological tumor characteristics [8]. Early studies have shown encouraging results [17]. Thus, in recent years, extraordinary efforts have been devoted to identifying predictive biomarkers’ response, some of which are already used in clinical settings for decision-making as BRAF and KIT mutation status for melanoma [21]. Circulating biomarkers are, therefore, of great interest because of their inexpensiveness, accessibility and non-invasive nature.

In this context, the aim of this study was to evaluate the immune response regulation during a high-dose hypo fractionated radiotherapy followed by a Nivolumab therapy in a patient with metastatic melanoma and to correlate biological data and clinical response. The goal was to highlight circulating immune biomarkers of response to treatments.

Overall, results showed lymphocyte activation after radiotherapy, followed by a decrease in activation during cancer relapse. However, after the introduction of a single injection the immune check point inhibitor (anti-PD1), patient immune status was increased in favor of an immune activation. Furthermore, the patient experienced adverse events after each treatment received indicating a systemic response. Vitiligo is an adverse effect correlated with a good response to immunotherapy [22]. Up to now, the patient presents a complete clinical response.

In this study, before treatment (T0), the patient has a rather favorable immune environment. Indeed, high immune subpopulation proportions within PBMCs are observed. nTregs and iTregs proportions are very low. Anti-tumor cytokines IFNy and IL-2 also have high values and the IFNy/IL10 ratio is greater than 2.

Nevertheless, after radiotherapy (T1) the different immune sub-population analysis reveals an immunosuppression. This immunosuppression is reversible because of immune sub-populations restoration until T4. For this patient, immune populations fall at T5. Interestingly, this status is clinically correlated with patient stability at T4 and patient clinical progress at T5.

There is also effective immune response establishment. Indeed, dendritic cells proportion (CD3-CD11c+CD14low/-) increases during H-SRT after T1. A transcriptomic analysis also showed an increase in DC maturation markers gene expression (CD80, CD83, CD86), which are witnesses of a previous antigen capture by DCs and their future maturation status (data not shown). LT CD4 and LT CD8 proportions increase also, even though there is small variation in activation markers. However, this immune response boost after radiotherapy seems transient, as a decrease in all these markers appears; it corresponds to the patient’s cancer progression. 

Radiation therapy is, therefore, responsible of an early activation of the immune system. This immune activation seems to correlate to the clinic regarding patient initial symptomatology (chills, vitiligo) as well as good local control. However, this activation is transient. Indeed, at T5 an unfavorable profile is observed with a decrease in most cell populations. After Nivolumab introduction between T5 and T6, there was an increase in all immune subpopulations, except for NK cells and DC, and more importantly for CD4+, CD14+ and CD19+. Immune system activation is supported by the analysis of activation markers, which increase after T5 for all studied ones.

iTregs increase at T6 could be explained by the tumor antigen release after anti-PD1 treatment. In addition, the need to restore cellular homeostasis and patient immune functions lead to nTregs increase in T8. However, during treatment, if many antigens are released then DCs will mature later and/or migrate to the nearest draining lymph nodes or mature during their course to secondarily activate the adaptive response.

Unexpectedly, a significant increase in B-cells after immunotherapy is observed. Nevertheless, it has been reported that PD1 is expressed at the lymphocyte level and it has been shown in a mouse model that the PD-1 antigen expression is tightly regulated and induced by signal transduction across the antigen receptor [23]. The possibility that the PD-1 antigen may play a role in clonal selection of B-cells is not excluded. Accordingly, this increase could also be related to the Th2 immune response observed after the introduction of Nivolumab with IL-4 and IL-6 cytokines.

Very interestingly, we showed for the first time that a patient’s exosomes secreted after H-SRT are in favor of an immune activation with an increase of the proliferation of immune cells while those secreted after a Nivolumab single dose did not activate or inhibit immune cell proliferation. This observation is quite interesting as it is the opposite of clinical observation. It seems to show that the good response after a Nivolumab injection is probably not mediated by exosomes. 

Immediately after immunotherapy introduction, an increase in T cell lymphocytes, CD14+ monocytes, LB and iTregs is observed. Nivolumab may also induce tolerogenic DCs. Nevertheless, additional markers, particularly of DC differentiation and maturation, have to be explored to validate this hypothesis. LB increase has not been described under immunotherapy as LBs have never been described as potential biomarkers of anti-PD1 immunotherapy efficacy. Nevertheless, the expression of PD1 by the LB could be an interesting element to explore. 

## 4. Conclusions

In conclusion, although we noted that this case study did not allow for the identification of an early biomarker of response to treatment, all results obtained in this exceptional case open an interesting therapeutic perspective and raise the question of the necessity of combining therapies to optimize the response to treatments. Indeed, it seems that the combination of H-SRT with anti-PD1 immunotherapy may favor an activation of the immune response which could result in a better clinical response. The results of this pilot study are very encouraging, but it should be validated on a larger cohort. The advent of immunotherapy has brought about a paradigm shift in the advanced cancer treatment landscape. These novel therapeutics may result in unconventional response patterns and, therefore, special strategies are needed for an accurate assessment of tumor responses and clinical outcomes. Development of robust biomarkers to identify responder patients and monitor immunotherapy response is a key step for further progress in this area and the development of precision immuno-oncology approaches. The circulating biomarkers analyzed in our study have the advantage of being easily evaluable in common tests used in clinics and are also suitable for serial sampling during treatment. However, given the complexity of tumor-host interactions and the dynamic nature of most immunologic biomarkers, it is not surprising that each of them has shown limited value when used individually. Efforts are needed to develop integrative models that consider multiple tumor and host parameters and characteristics, such as the “cancer immunogram” [9]

In this precision medicine era, predictive biomarkers are becoming critical to treatment decisions, but this component is not sufficiently integrated in most clinical studies. Future studies involving the systematic and prospective collection of matched blood and tumor samples are needed to confirm these signatures in larger and multicenter cohorts. A predictive signature could then be used directly in clinical practice to stratify patients before starting immunotherapy.

## Figures and Tables

**Table 1:** Flow cytometry panel with antibodies and corresponding isotypes control.

Panel	Antibodies	Isotypes
Cell type	CD4 (VIT4)-VioBlue	Mouse IgG2a-VioBlue
	CD8-VioGreen	Mouse IgG2a-VioGreen
	CD19-VioBright FITC	Mouse IgG1-VioBright FITC
	CD14-PE	Mouse IgG2a-PE
	CD3-PE-Vio770	Mouse IgG2a-PE-Vio770
	CD335 (NKp46)-APC	Mouse IgG1-APC
	CD11c-APC-Vio770	Mouse IgG2b-APC-Vio770
Activation	CD4 (VIT4)-VioBlue	Mouse IgG2a-VioBlue
	CD30-APC-Vio770	Mouse IgG1-APC-Vio770
	CD69-PE-Vio770	Mouse IgG1-PE-Vio770
	Anti-HLA-DR-PerCP-Vio700	Mouse IgG2a-PerCP-Vio770
	CD152-APC	Mouse IgG2a-APC
	CD197 (CCR7/REA108)-PE	REA Control (S)-PE
	CD25-VioBright FITC	Mouse IgG2b-VioBright FITC
	CD8-VioGreen	Mouse IgG2a-VioGreen
nTregs	CD4 (VIT4)-VioBlue	Mouse IgG2a-VioBlue
	CD25-VioBright FITC	Mouse IgG2b-VioBright FITC
	CD127-PE-Vio770	Mouse IgG2a-PE-Vio770
iTregs	CD4 (VIT4)-VioBlue	Mouse IgG2a-VioBlue
	CD18-FITC	Mouse IgG1-FITC
	CD223-PE	REA Control (S)-PE
	CD49b-PE-Vio770	REA Control (S)-PE-Vio770
	CD152-APC	Mouse IgG2a-APC
